# A Simple RP-HPLC Method for Quantitation of Itopride HCl in Tablet Dosage Form

**DOI:** 10.4103/0975-1483.71634

**Published:** 2010

**Authors:** Rajan VS Thiruvengada, Saleem TS Mohamed, S Ramkanth, M Alagusundaram, K Ganaprakash, Chetty C Madhusudhana

**Affiliations:** *Department of Pharmaceutical Analysis, Annamacharya College of Pharmacy, Rajampet - 516 126, India*; 1 *Department of Pharmacology, Annamacharya College of Pharmacy, Rajampet - 516 126, India*; 2 *Department of Pharmaceutics, Annamacharya College of Pharmacy, Rajampet - 516 126, India*

**Keywords:** Dosage formulation, itopride HCl, method validation, RP-HPLC, UV detection

## Abstract

An isocratic reversed phase high-performance liquid chromatographic method with ultraviolet detection at 220 nm has been developed for the quantification of itopride hydrochloride in tablet dosage form. The quantification was carried out using C_8_ column (250 mm × 4.6 mm), 5-μm particle size SS column. The mobile phase comprised of two solvents (Solvent A: buffer 1.4 mL *ortho*-phosphoric acid adjusted to pH 3.0 with triethyl amine and Solvent B: acetonitrile). The ratio of Solvent A: Solvent B was 75:25 v/v. The flow rate was 1.0 mL ^-1^with UV detection at 220 nm. The method has been validated and proved to be robust. The calibration curve was linear in the concentration range of 80-120% with coefficient of correlation 0.9995. The percentage recovery for itopride HCl was 100.01%. The proposed method was validated for its selectivity, linearity, accuracy, and precision. The method was found to be suitable for the quality control of itopride HCl in tablet dosage formulation.

## INTRODUCTION

Itopride hydrochloride (*N*-[4-[2-(dimethylamino)-ethoxy] benzyl]-3,4-dimethoxybenzamide hydrochloride) is a novel gastroprokinetic agent, which stimulates gastrointestinal motor activity through synergistic effects of dopamine D2 receptor blockade and acetylcholine esterase inhibition.[1,2] Itopride HCl is prescribed for the gastrointestinal symptoms caused by reduced gastrointestinal mobility, e.g., a feeling of gastric fullness, upper abdominal pain, anorexia, heartburn, nausea and vomiting, caused by conditions such as functional dyspepsia or chronic gastritis. The extraction reported to detect itopride HCl was liquid-liquid extraction.[3–5] However, this method presented some disadvantages such as being of low sensitivity, time consuming, and costly. They were reported for the determination of itopride HCl and its related substances in biological fluids like plasma, blood, and urine only but, not a single method has been reported for its determination in bulk and solid (tablet) dosage forms by reversed phase high-performance liquid chromatographic (RP-HPLC) method. This study was designed to develop a simple and reliable method to quantitate itopride HCl in a relatively short time with high linearity. Therefore, this study focused on the development of simple and rapid isocratic RP-HPLC method which can be employed for the routine analysis of itopride HCl in bulk drug and formulations. The established method was validated with respect to specificity, linearity, precision, accuracy, and ruggedness.

## MATERIALS AND METHODS

### Chemicals and reagents

All chemicals and reagents were of HPLC grade quality. Itopride HCl was a gift sample from Micro Labs Limited, Hosur, Tamilnadu, India.

### Instrumentation and chromatographic conditions

An HPLC system consisted of Schimadzu UV detector, C8 (5 μm, 250 mm × 4.6 mm) column was used for separation and the chromatograph were recorded using CLASS-VP software. The separation was carried out under isocratic elution with mobile phase was a mixture (75 volumes) of 1.4 mL of *ortho*-phosphoric acid in 1000 mL of water and adjust the pH 3.0 by using triethyl amine and acetonitrile (25 volumes), was filtered through 0.4 μm nylon membrane filter before use. The flow rate was 1.0 mL min^-1^, the analyte was monitored at 220 nm, and the injection volume was 20 μL.

### Standard and sample preparation

A standard stock solution of 50 mg of itopride HCl in mobile phase was prepared in a volumetric flask. From this stock solution, about 10 mL was diluted to 100 mL with mobile phase.

### Estimation of itopride HCl in tablet dosage form

Twenty tablets were weighed and crushed to a fine powder. The powder equivalent of 50 mg of itopride HCl was taken in a 100-mL volumetric flask containing mobile phase and kept sonication for 10 min and made up to mark with mobile phase. The resultant mixture was filtered through 0.45 μm nylon filter. The desired concentration for the drug was obtained by accurate dilution, and the analysis was followed up as in the general analytical procedure.

## RESULTS AND DISCUSSION

### Development and optimization of isocratic HPLC conditions

**Figure 1:**
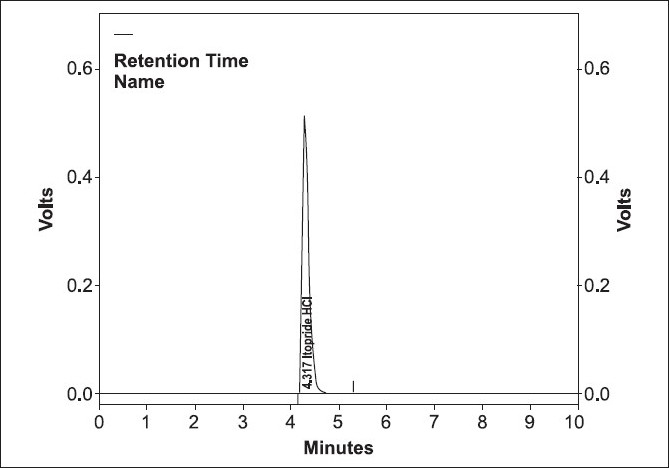
Chromatogram of Itopride HCl

**Figure 2:**
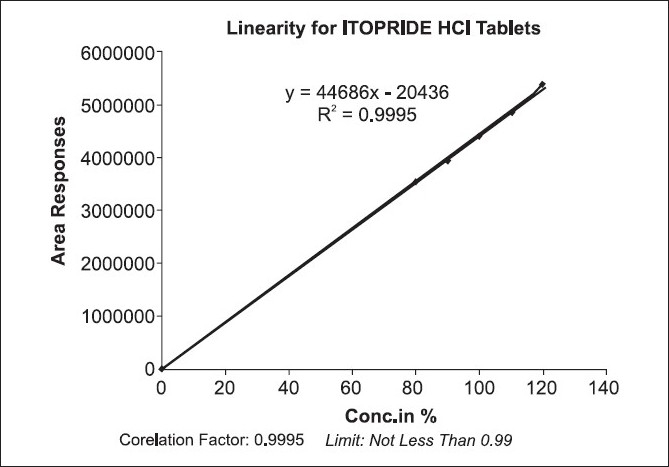
Linearity graph for Itopride HCl

A UV scan of itopride HCl showed a maximal absorbance at or near 220 nm. Initial method development was conducted on a C8 (5 μm, 250 mm × 4.6 mm) column was used for separation at ambient temperature. The chromatographic conditions were optimized with respect to specificity, resolution, and time of analysis. The results of the chromatogram were retention time = 4.217, USP tailing factor = 1.44, theoretical plate no. = 6144.7, calibration range = 80-120%, and area = 4437618. Linearity was studied by preparing standard solution at different concentration levels. The linearity range was found to be 80-120%. The regression equation was found to be *y* = 44686*x* with coefficient of correlation 0.9995 where *x* is concentration and *y* is peak area.[6] A mobile phase of a mixture (75 volumes) of 1.4 mL of *ortho*-phosphoric acid in 1000 mL of water and adjust the pH 3.0 by using triethyl amine and acetonitrile (25 volumes), was found to provide a reproducible, baseline resolved peak [Figures 1 and 2]. These conditions allowed for separation of itopride HCl from tablet formulation.

### Method validation

For validation of analytical methods, the guidelines of the International Conference on the Harmonization of Technical Requirements for the Registration of Pharmaceuticals for Human Use (ICH 1996) and (USP 2003) have recommended the accomplishment of accuracy tests, precision, specificity, linearity, ruggedness, and robustness of the method.

### Intra- and interday accuracy precision

Precision was studied to find out intra- and interday variations in the test methods of itopride HCl at six different weight levels 304.4, 305.6, 308.2, 299.1, 305.6, and 300.1 mg two times same day and different day, respectively. The percentage RSD was calculated which should be <2%. Intraday precision was done on the same day and the percentage RSD was calculated, and interday precision was done on the different day and percentage RSD was calculated. The results were shown in Tables 1 and 2.

**Table 1:** Intraday precision characteristics of itopride recommended for the HCl

Weight of samples (g)	Injection volume (μL)	Mean area	RSD (%)
304.4	20	4429594	0.03
305.6	20	4462525	0.59
308.2	20	4568540	0.23
299.1	20	4319730	0.11
305.6	20	4395803	0.04
300.1	20	4322305	0.01

**Table 2:** Interday precision characteristics of itopride HCl

Weight of samples (g)	Injection volume (μL)	Mean	RSD (%)
304.1	20	4446587	0.40
303.7	20	4453466	0.19
307.9	20	4548451	0.00
300.3	20	4333103	0.09
302.7	20	4397236	0.14
304.1	20	4332490	0.40

### Accuracy

Accuracy was determined by recovery study of itopride HCl known amount of standard itopride HCl was added into preanalyzed sample and subjects them to the proposed HPLC method. The results of recovery studies are shown in Table 3. The study was carried out at three different concentration levels.[7]

**Table 3:** Recovery studies of itopride HCl

Labeled amount (mg)	Amount added (mg)	Amount recovered (mg)	% Recovery
50.0	40.40	40.38	99.95
50.0	50.90	51.30	100.79
50.0	60.10	59.68	99.29

### Specificity

Subjecting the drug solution in different stress conditions such as acid, base, and peroxide, and the degradation was noted.[8,9] The results were shown in Table 4.

**Table 4:** Recovery studies of itopride HCl

Specificity	Weight of sample (g)	Time (h)	RT of itopride HCl	RT of degraded product
Acid stress (0.5 N HCl)	0.305	0	4.300	4.308
		8	4.301	4.310
Base stress (5 N NaOH)	0.305	0	4.325	4.317
		8	4.322	4.314
Peroxide stress (3% H_2_O_2_)	0.305	0	4.233	4.217
		8	4.244	4.221

### Robustness

This was done by small deliberate changes in the chromatographic conditions at three different levels -1, 0, +1, and retention time of itopride HCl was noted. The factors selected were flow rate, pH, and % acetonitrile in the mobile phase. Results shown in Table 5 indicate that the selected factors remained unaffected by small variations of these parameters.

**Table 5:** Robustness characteristics of itopride HCl

Factor	Level	Retention time
Flow rate (mL/min)		
0.9	-1	4.675
1.0	0	3.833
1.1	+1	3.825
pH of mobile phase		
2.9	-1	3.667
3.0	0	3.675
3.1	+1	4.808
Percentage acetonitrile in the mobile phase		
22.5	-1	3.800
25.0	0	3.792
27.5	+1	5.233

## CONCLUSION

A simple and reliable HPLC method for measuring itopride HCl in pharmaceutical dosage formulation has been developed. A fully validated RP-HPLC procedure for the assay of itopride HCl drug in tablet formulation is described for the first time. Hence, it can be recommended for the routine quality control of this drug. The simplicity of the HPLC procedure, the short run time, and the low volume of injection make this method suitable for quick and routine analysis. The intraday run and interday run variability and accuracy results were with in the acceptable limit.
